# Supplementary material for: Linking Gba1 E326K mutation to microglia activation and mild age-dependent dopaminergic Neurodegeneration
Source: bioRxiv. 2023 Sep 14:2023.09.14.557673. Preprint. [Version 1] doi: 10.1101/2023.09.14.557673 (PMC10515932; doi:10.1101/2023.09.14.557673)
Supplement: Supplement 1 [file media-1.pdf]

BioRvix
Supplementary information

Linking *Gba1* E326K mutation to microglia activation and mild age-dependent dopaminergic Neurodegeneration

Sin Ho Kweon[1,2,*], Hye Guk Ryu[3,*], Hyeonwoo Park[3], Saebom Lee[1,2,4], Namshik Kim[1,2], Seung-Hwan Kwon[1,2], Shi-Xun Ma[1,2], Sangjune Kim[3,#], and Han Seok Ko[1,2,5,#]

[*,#]These authors contributed equally to this work.

Correspondence: sangjune@chungbuk.ac.kr (S.K.) or hko3@jhmi.edu (H.S.K)

This file includes

Supplementary Figure 1 to 5 and Figure legends

Supplementary Table 1

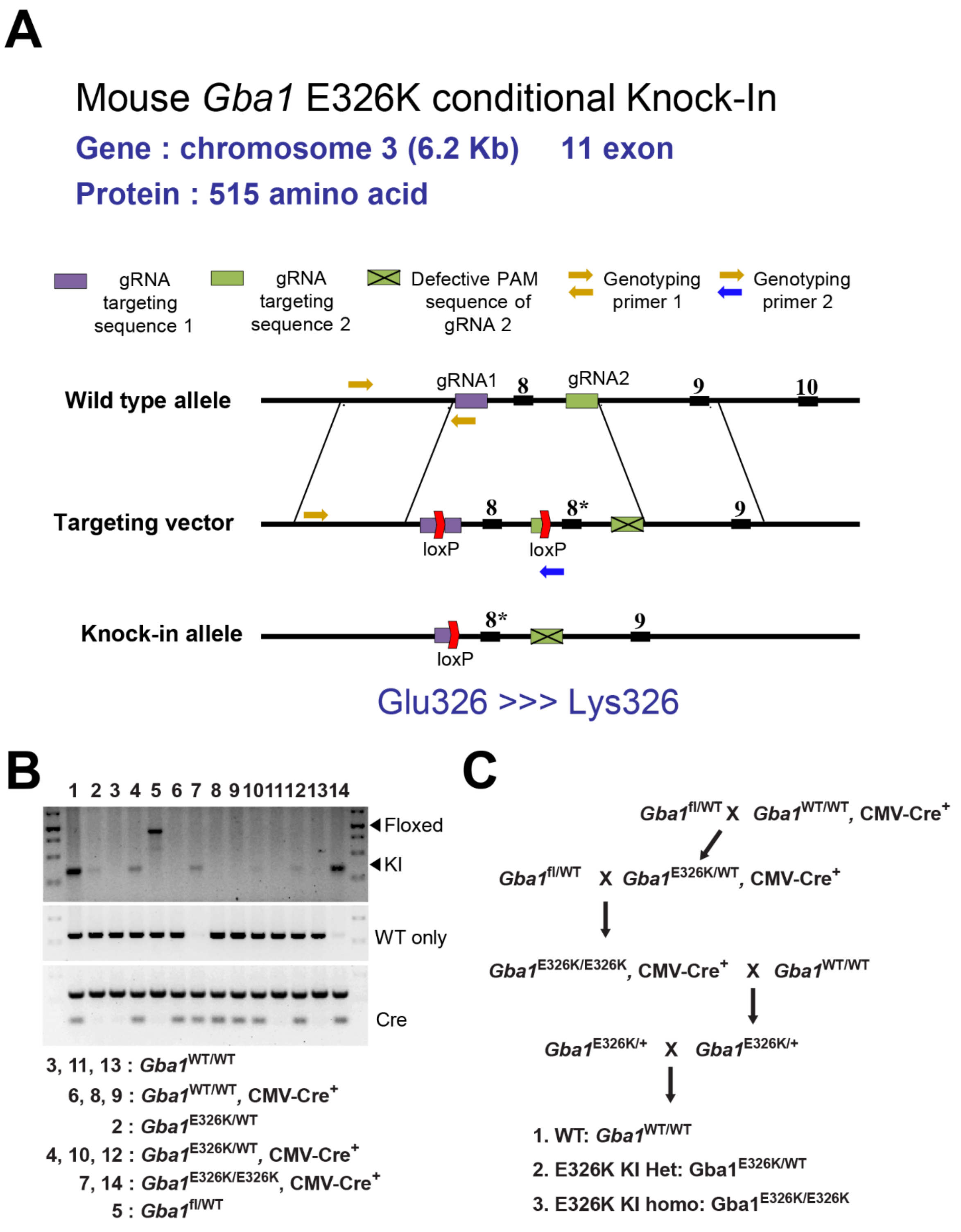

Figure S1. Generation of E326K *Gba1* conditional KI mice. *E326K Gba1* conditional KI mice were generated by homologous recombination using a targeting vector containing exon 8 flanked by loxP sites, followed by the mutated exon 8 (g1030G>A, Glu326Lys) and two sgRNA (1st sgRNA: 5'-gggccctggaaagttcagagtgg-3', 2nd sgRNA: 5'-tgtgaaagagaagataaccatgg-3'). Mice were then crossed with CMV-Cre transgenic mice to generate *E326K Gba1* mice. (A) Diagram illustrating the strategy for targeting E326K conditional KI mice. (B) Genotyping. (C) Breeding strategy.

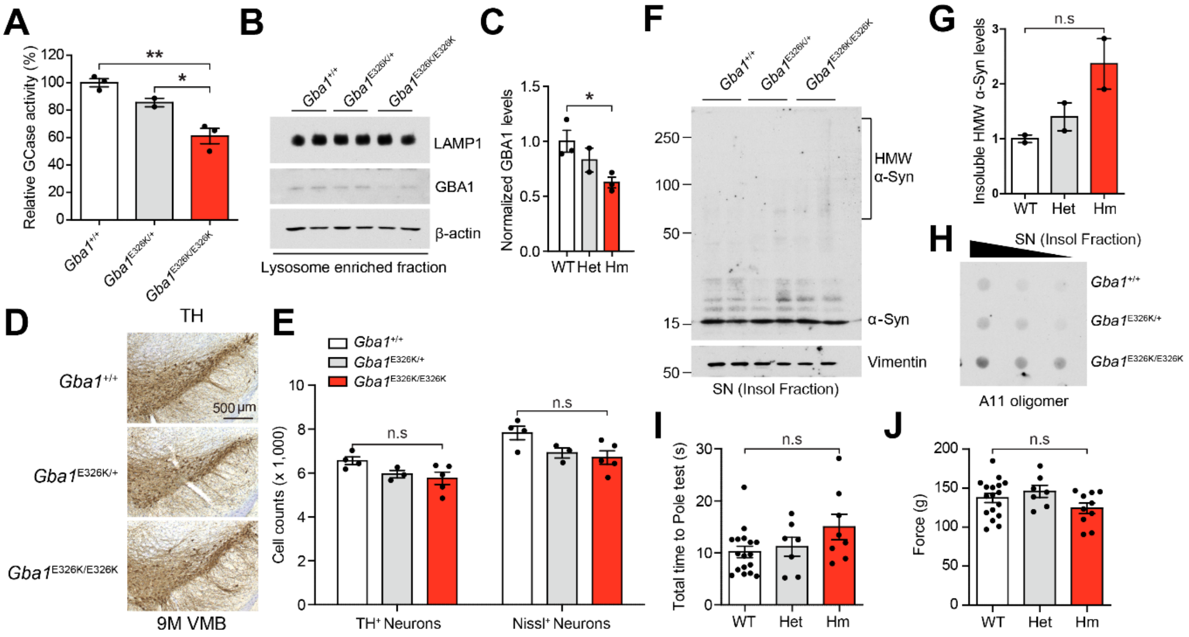

Figure S2. Characterization of E326K *Gba1* KI mice. (A) GBA1 enzyme activity in the ventral midbrain of 9-month-old WT, Het, and Hm of E326K *Gba1* KI mice (n=2-3). (B) GBA1 protein levels were assessed using Western blot analysis in the lysosome-enriched fraction from the ventral midbrain. (C) Quantification of normalized GBA1 protein levels (n=2-3). (D) Representative photomicrographs of coronal mesencephalon sections containing TH-positive neurons in the SNc region. (E) Stereology counts of TH- and Nissl-positive neurons in the SNc region. Unbiased stereologic counting was performed in the SNc region (n=3-5). (F) Representative immunoblot for α-Syn in the Triton X-100 insoluble fraction. (G) Quantification of insoluble HMW α-Syn levels (n=2). (H) Dot blot assay with anti-A11 oligomer antibody in the Triton X-100 insoluble fraction of the ventral midbrain. (I-J) Results of mice on the (I) pole test, and (J) forelimb grip strength test (n=7-17). The error bars represent the S.E.M. *$P < 0.05$, **$P < 0.01$. n.s., not significant.

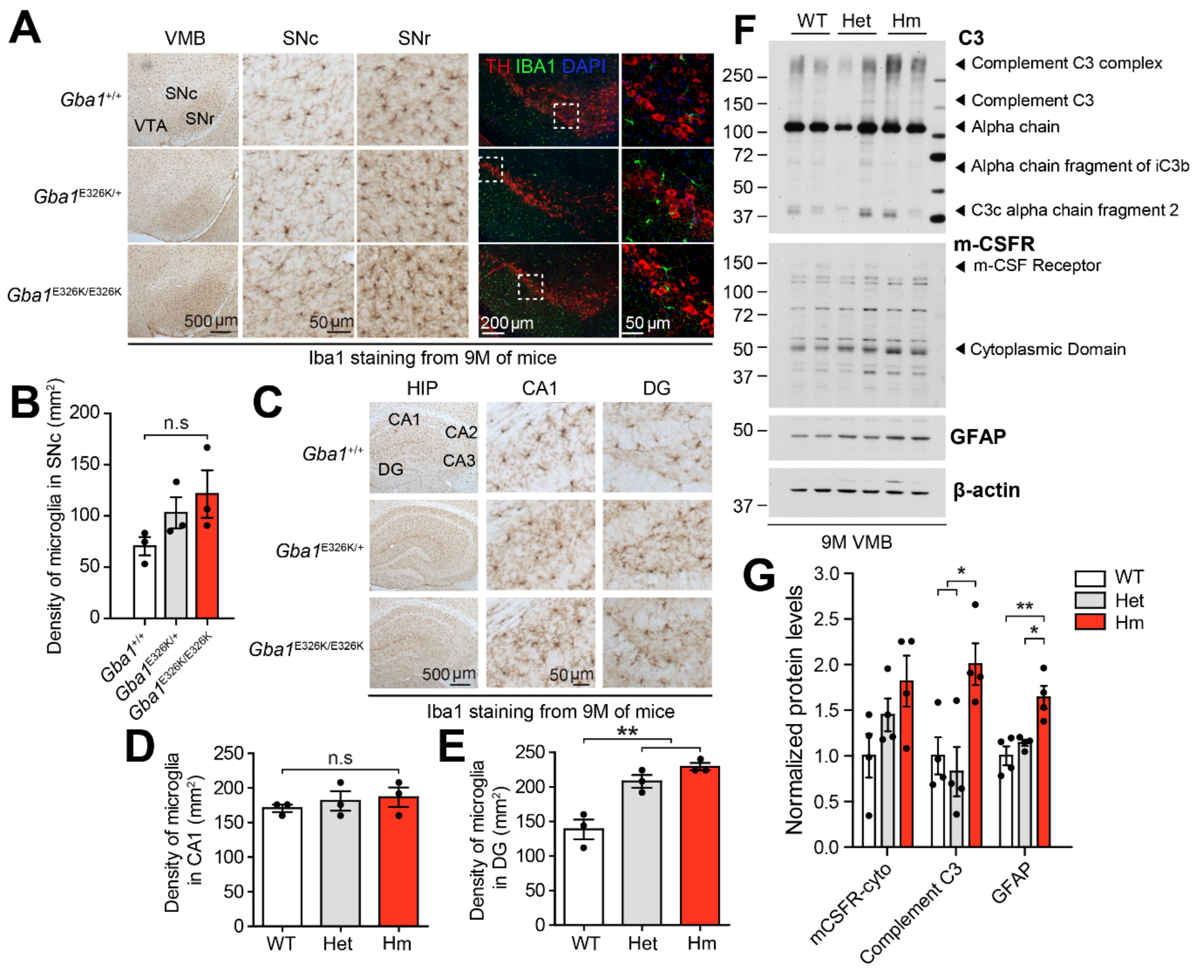

Figure S3. Microglia activation in E326K *Gba1* KI mice at 9 months of age. (A) Representative photomicrographs of coronal mesencephalon sections containing Iba1-positive microglia and immunostaining for Iba1 (Green) and TH (red) in the SNc region. (B) Quantification of microglia density in the SNc (n=3). (C) Representative photomicrographs of coronal mesencephalon sections containing Iba1-positive microglia in the hippocampus. (D) Density of microglia in the CA1 and (E) in the dentate gyrus (n=3). (F) Representative immunoblots for C3, m-CSFR and GFAP in the SNc region. (G) Quantification of normalized protein levels of m-CSFR-cytoplasmic domain, complement C3, and GFAP (n=4). The error bars represent the S.E.M. *$P < 0.05$, **$P < 0.01$. n.s., not significant.

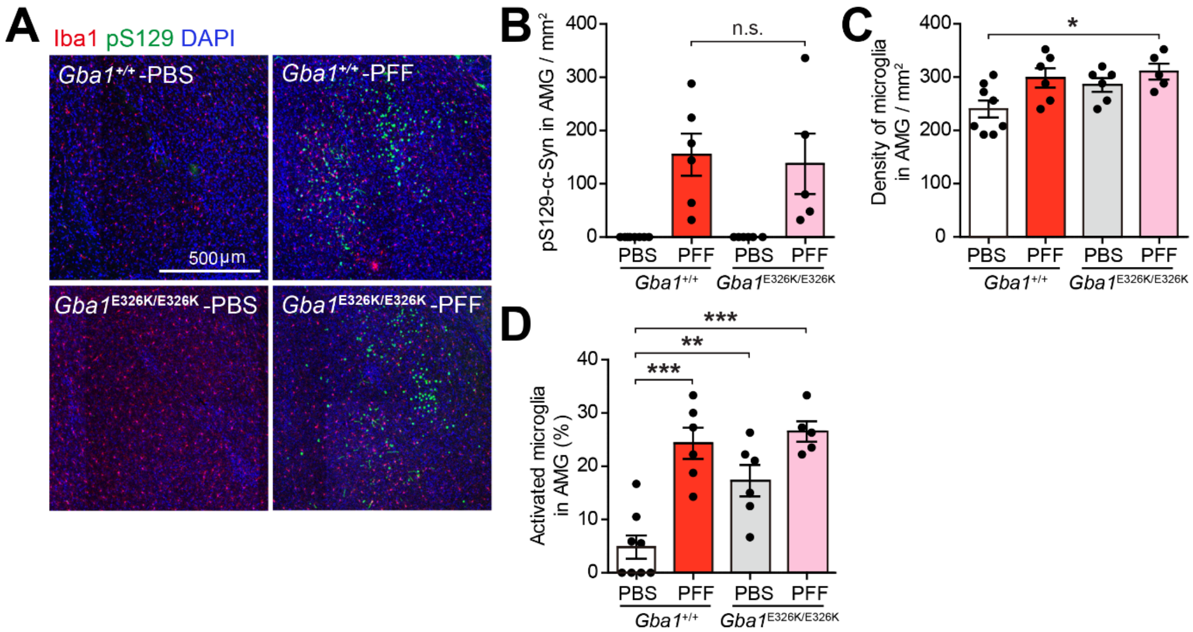

Figure S4. α-Syn pathology and neuroinflammation in the amygdala of E326K *Gba1* KI mice induced by α-syn PFF injection into the gut. (A) Representative immunostaining for pS129-α-syn (green) and Iba1 (red) in the amygdala region 7 months post-injection. (B) Quantification of the number of pS129-α-syn in the basolateral amygdala. (C) Quantification of the number of microglia in the basolateral amygdala. (D) Percentage of activated microglia in the basolateral amygdala (n=5-8). Error bars represent the mean ± S.E.M. *$P < 0.05$, **$P < 0.01$, ***$P < 0.001$. n.s., not significant.

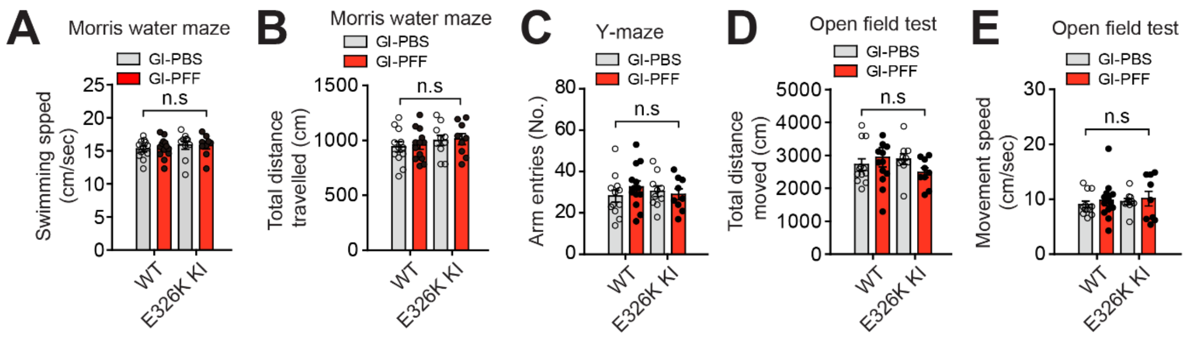

Figure S5. Additional results for non-motor symptoms in E326K *Gba1* KI mice induced by α-syn PFF injection into the gut. (A) Swimming speed and (B) total distanced traveled in probe trial sessions of the Morris water maze test (n=9-13). (C) Number of arm entries in the Y-maze test (n=9-13). The data of (D) total distance moved and (E) movement speed in the open-field test (n=9-13). Error bars represent the mean ± S.E.M.

Table S1. Primers used in this study

Gene	Primer Sequence
Tnfa	F; CCCTCACACTCAGATCATCTTCT
	R; GCTACGACGTGGGCTACAG
Il1a	F; GCACCTTACACCTACCAGAGT
	R; AAACTTCTGCCTGACGAGCTT
Il1b	F; GCAACTGTTCCTGAACTCAACT
	R; ATCTTTTGGGGTCCGTCAACT
Il6	F; TAGTCCTTCCTACCCCAATTTCC
	R; TTGGTCCTTAGCCACTCCTTC
Lcn2	F; CCAGTTCGCCATGGTATTTT
	R; CACACTCACCACCCATTCAG
Steap4	F; CCCGAATCGTGTCTTTCCTA
	R; GGCCTGAGTAATGGTTGCAT
S1pr3	F; AAGCCTAGCGGGAGAGAAAC
	R; TCAGGGAACAATTGGGAGAG
Timp1	F; AGTGATTTCCCCGCCAACTC
	R; GGGGCCATCATGGTATCTGC
Hspb1	F; GACATGAGCAGTCGGATTGA
	R; GGATGGGGTGTAGGGGTACT
Cxcl10	F; CCCACGTGTTGAGATCATTG
	R; CACTGGGTAAAGGGGAGTGA
H2-T23	F; GGACCGCGAATGACATAGC
	R; GCACCTCAGGGTGACTTCAT
Serping1	F; ACAGCCCCCTCTGAATTCTT
	R; GGATGCTCTCCAAGTTGCTC
H2-D1	F; TCCGAGATTGTAAAGCGTGAAGA
	R; ACAGGGCAGTGCAGGGATAG
Ggta1	F; GTGAACAGCATGAGGGGTTT
	R; GTTTTGTTGCCTCTGGGTGT
Ligp1	F; GGGGCAATAGCTCATTGGTA

	R; ACCTCGAAGACATCCCCTTT
Gbp2	F; GGGGTCACTGTCTGACCACT
	R; GGGAAACCTGGGATGAGATT
Fbln5	F; CTTCAGATGCAAGCAACAA
	R; AGGCAGTGTCAGAGGCCTTA
Clcf1	F; CTTCAATCCTCCTCGACTGG
	R; TACGTCGGAGTTCAGCTGTG
Tgm1	F; CTGTTGGTCCCGTCCCAAA
	R; GGACCTTCCATTGTGCCTGG
Ptx3	F; AACAAGCTCTGTTGCCCATT
	R; TCCCAAATGGAACATTGGAT
S100a10	F; CCTCTGGCTGTGGACAAAAT
	R; CTGCTCACAAGAAGCAGTGG
Sphk1	F; GATGCATGAGGTGGTGAATG
	R; TGCTCGTACCCAGCATAGTG
Cd109	F; CACAGTCGGGAGCCCTAAAG
	R; GCAGCGATTTCGATGTCCAC
Ptgs2	F; GCTGTACAAGCAGTGGCAAA
	R; CCCCAAAGATAGCATCTGGA
